# Brain Magnetic Resonance Imaging Findings in Developmentally Delayed Children

**DOI:** 10.1155/2011/386984

**Published:** 2011-11-02

**Authors:** Ali Akbar Momen, Gholamreza Jelodar, Hamid Dehdashti

**Affiliations:** ^1^Department of Pediatric Neurology, Golestan Hospital, Ahvaz Jundishapur University of Medical Sciences, P.O. Box 173-61355, Ahvaz, Iran; ^2^MRI Ward, Department of Radiology, Golestan Hospital, Ahvaz Jundishapur University of Medical Sciences, P.O. Box 173-61355, Ahvaz, Iran

## Abstract

*Background*. Developmental disorders are failure or inability to acquire various age-specific skills at expected maturational age, which affects about 5–10% of preschool children. One of the most important methods for evaluation of developmentally delayed children is neuroimaging, especially, brain magnetic resonance imaging (MRI) that provides useful information regarding brain tissue structures and anomalies. *Method and Material*. In this study, hospital records of 580 developmentally delayed children (aged 2 months to 15 years) who admitted in pediatric ward of Golestan Hospital from 1997 to 2009 were selected. Information such as age, MRI findings were collected in the questionnaire and statistically analyzed. *Results*. Total, 580 children including 333 males (57.4%) and 247 females (42.6%) were studied. Abnormal brain MRI was observed in 340 (58.6%) cases (204 Males, 136 females). The finding includes nonspecific in 38 (6.6%), congenital and developmental anomalies of brain in 39 (6.7%), recognizable syndromes in 3 (0.5%), neurovascular diseases or trauma in 218 (37.6%), and metabolic or neurodegenerative diseases in 42 (7.2%) cases. *Conclusion*. Because 60% of all study groups showed abnormal brain MRI, using this method could be effective in diagnosis, management, and almost prognosis determination processes.

## 1. Introduction

Development is a complex and continuous process of maturity, parallel to the growth of children, which can affect many aspects, and begins from conception and continues until maturity [1]. During the development, an infant can establish a variety of relations with their surrounding environment. Unlike the sequence, the rate of development varies from a child to another and depends on genetic factors, environmental aspects, and chronic diseases [2]. Development in pediatrics will be evaluated based on four domains of gross motor, fine motor, and social and language skills. Children who cannot gain/acquire appropriate developmental skills at the expected age have been considered suffering from developmental delay.

Global developmental delay (GDD) is a subset of developmental disorders that is defined as a significant delay or below the appropriate standard in two or more developmental domains. It may occur due to static or progressive disorders in the central nervous system. In the patients with these disorders, regression, stability, or disease progression may develop. The main causes of delay in development include a range of various diseases from which the large number associate with specific findings in brain MRI. There are various methods and tests to evaluate the development. The most prestigious test is Denver Developmental Screening Test (DDST) and its modified form is Denver Developmental Screening Test II (DDSTII) [3]. 

These causes cannot be identified only based on physical examination or patient history; however, additional studies like genetic analysis, metabolic, serological, strip brain, and neuroimaging are required. Neuroimaging provides important information as evidence of previous injuries, specific abnormalities that could indicate a group or a particular disease [1, 4, 5]. 

Prevalence of developmental delay in children has been reported 5–10% [6]. Brain MRI is one of the major evaluations of patients, and based on previous studies, about 60% of cases has abnormal brain MRI [7]. A complete study has not been done so far. As this type of study has not been done especially in the Khuzestan province, it will provide useful information about the patients, the rate, and type of brain abnormalities. It also helps to identify these diseases informing the parents and preventing the recurrence of similar cases. On the other hand, it may remind the authorities about the necessity of right culture and suitable planning when the cost of diagnostics and care of patients is very high.

The aim of this study was to determine the relative prevalence of abnormal brain MRI, as the most important method for brain disorder diagnosis, in GDD children aged 2 months to 15 years.

## 2. Materials and Methods

### 2.1. Patients, Method, and Data Collection

In this study, 580 patients with GDD who have been referred for diagnosis to children neurology ward in Golestan Hospital from the beginning of 1997 until the end of 2009 were selected. The patients have been studied based on clinical, para-clinical information and particularly brain MRI findings, then the information extracted and analyzed. The findings presented in the MRI reports were divided in six groups as described previously [1]: (1) normal; (2) non-specific findings, including cavum septum pellucidum, cavum vergae, ventriculomegaly, enlarged subarachnoid spaces, hypoplasia of the corpus callosum, and delayed myelination (3) congenital and developmental abnormalities of the brain, that are divided as three general groups; (4) recognisable syndromes such as neurofibromatosis, tuberous sclerosis, Sturge-Weber syndrome; (5) Neurovascular diseases and trauma including hypoxic-ischaemic injury or encephalopathy, periventricular leukomalacia, encephalomalacia, atrophy, and gliosis; (6) metabolic and neurodegenerative diseases such as Demyelination. The Information has been extracted based on the desired variables and recorded at a certain form that is provided for this purpose.

### 2.2. Inclusions Criteria

The patients who had GDD, aged between 2 months and 15 years, admitted for the first time to diagnose the cause of delay, and have brain MRI were selected.

### 2.3. Exclusion Criteria

The children with GDD who have been admitted without brain MRI had been excluded. Furthermore, the children who have been admitted several times for various reasons have been considered only once.

### 2.4. Statistical Analysis

The needed information has been gathered from all eligible patients and recorded in particular forms that were designed for this purpose. Then, the data were analyzed using SPSS 18 Software. Frequency was described and for evaluating any association between qualitative variables was observed using Chi-square test. The *P* value less than 0.05 considered as significant difference.

## 3. Results

**Figure 1:**
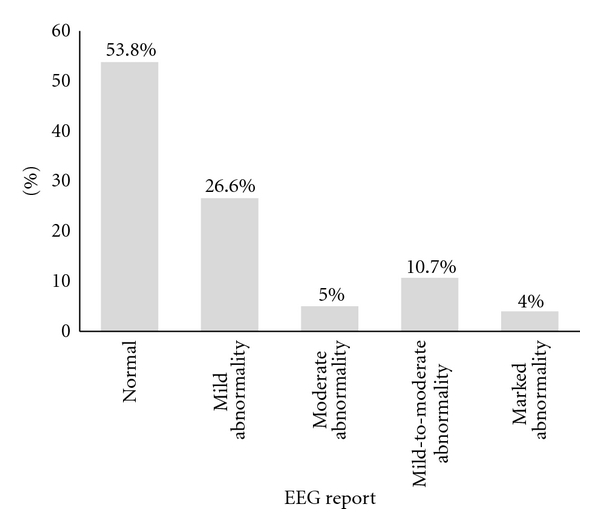
Percentage classification of different disorders in electroencephalography report among study groups.

In this study, 580 children with developmental disorders were studied in a comprehensive number of 333 (57.4%) males and 247 (42.6%) females. Patients are divided in four age groups: 2 to 6 months, 7 months to 2 years, 2 to 5 years, and more than 5 years. Brain MRI findings in 240 cases (41.4%) reported normal and in the rest 340 cases (58.6%) showed abnormal pattern. Among 340 abnormal MRI cases, 218 (37.6%) had neurovascular and trauma diseases. The relative frequencies of MRI findings in different age groups as well as gender were related mostly to the neurovascular and trauma diseases. From 273(47%) children aged 7 to 24 months, 114 (41.8%) cases were related to the neurovascular and trauma group, and also from 109 (19%) children aged 61 to 164 months, 32 ones (29.4%) were in this group. Also from the 333 male patients, 137 ones (41.1%) and, from 247 female, 81 ones (32.8%) were in this group (*P* = 0.332). About the gender relation between normal and abnormal MRI, results reported that both groups of male and female have abnormal MRI meaning from 333 patients, 204 males (61.3%) and from 247 females 136 cases (55.1%) showed abnormal MRI (*P* = 0.147) (Table 1). In case of electroencephalography, the mild abnormality was the most frequent one (26.6%) followed by mild-to-moderate abnormality (10.7%) (Figure 1).

**Figure 2:**
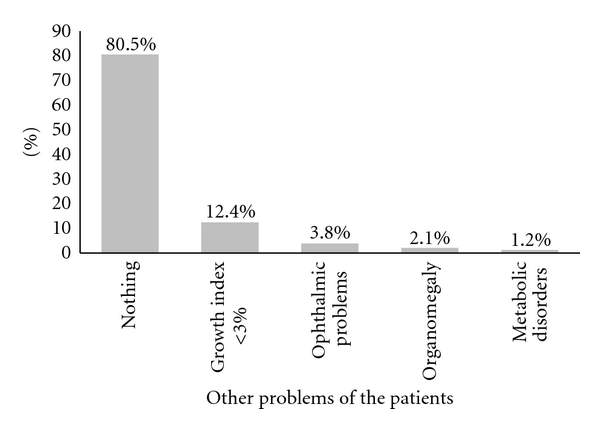
Percentage classification of different disorders in study groups.

**Figure 3:**
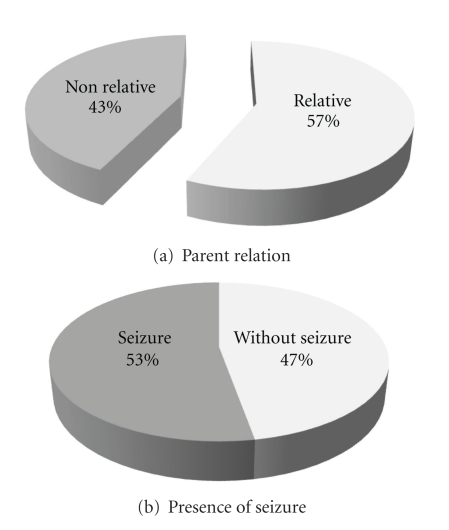
The classification of patients based on the relativity (a) and presence of seizure (b).

Another case where this study was considered was the birth history. In 336 cases (57.9%), a positive biography complication at birth or thereafter was noted meaning that 177 cases (30.4%) showed history of neonatal jaundice and 24 cases (4.1%) were associated with exchange transfusion. 274 cases (47.2%) did not have the seizure and 306 cases (52.8%) were reported to have seizure disorders, including different types of seizures. Parents of 330 patients (56.9%) were relatives and parents of 250 ones (43.1%) were nonrelated. According to the presence of associated disorders, 467 cases (80.5%) did not report abnormal findings on physical examination. 72 cases (12.4%), showed low growth indexes also 12 (2.1%) organomegaly, 22 (3.8%) eye problems, and 7 (1.2%), metabolic abnormalities was mentioned (Figure 2). According to other disorders evaluated and normal or abnormal MRI from 467 children without disorders, 273 ones (58.5%) and 113 children with disorders listed, 67 cases (59.3%) had abnormal MRI (Table 1) (*P* = 0.91). In evaluating normal and abnormal MRI based on the presence or absence of parents' consanguinity, 330 cases had relative parents and from those 201 ones (60.9%) had abnormal MRI. This finding in 250 cases with nonrelative parents was 139 (55.6%) cases (*P* = 0.2) (Figure 3(a)). According to presence or absence of seizures in 306 cases (52.8%) with GDD, seizure disorder also had been reported, in which 184 cases (60.1%) had abnormal MRI. The rest 274 patients had no seizures, of which 156 cases (56.9%) showed abnormal MRI (*P* = 0.44) (Figure 3(b)).

## 4. Discussion

GDD is the relatively common disease that affects 1% to 3% of children under five years. For these patients, history reviews and clinical examination are highly preferred to laboratory examination. Based on important data in their history and clinical findings, they may require various evaluations such as metabolic diseases, chromosomal abnormalities, and assessment of lead pollution. Neuroimaging routinely is recommended for these children especially if any disorders observed in their clinical examination, and brain MRI is preferred to CT-scan. We studied 580 brain MRI of children with GDD, while we tried to examine the history and physical examination findings that could be important. The study found 340 cases (58.6%) with development delay who had abnormal brain MRI. Statistically, differences were reported in the various studies such as Bouhadiba et al. [8] who conducted a study in France between 1997–1991 on 224 children with developmental delay and observed 109 cases (48.6%) with positive findings in brain MRI from whom 55 cases had structural anomalies of the brain. Another study in Korea between 1993–1991 on 34 children with GDD had been detected 26 cases (76.5%), with significant abnormal findings on brain MRI [9]. As in this study notable cases have been evaluated obtaining statistics intermediate between these figures seem to be logical.

Most findings in the brain MRI of these children have been related to the neurovascular and trauma disease group so 218 cases (37.6%) were observed in this group. Noting that 336 cases of patients (57.9%) reported positive history of problems around delivery such as asphyxia and neonatal jaundice, we may suggest some relations between these problems and complications occurring in the form of brain MRI disorders. We did not observe a gender significant difference between male and female in frequency of normal and abnormal brain MRI so that 129 (38.7%) from 333 male patients had normal and 204 ones (61.3%) had abnormal brain MRI. From 247 females, 111 (44.9%) had normal and 136 cases (55.1%) had abnormal brain MRI. According to the calculated *P* = 0.147, significant difference did not exist. We observed more normal brain MRI frequency in elder patients whereas the younger cases had higher abnormal brain MRI so that from 56 children aged between 2 and 6 months, 40 (70.59%) had abnormal while from 109 children aged 61–164 months, 47 patients (43.12%) had abnormal brain MRI (*P* = 0.008). One of the causes of this result is related to the changes that can be seen in the brain MRI of younger children and may be considered as positive findings while regarding age and clinical examination those findings are normal. So in the later follow-up and brain MRI, abnormal findings will not be seen.

This study showed that there are no distinguished differences between the relative frequency of GDD patients who suffer from seizures and those who do not suffer, and electroencephalograph (EEG) in 53.8% of them has been reported normal. On the other hand, seizures frequency was higher in children who had abnormal brain MRI than others who had normal brain MRI. So from 306 cases, patients with seizures were 184 (60.1%) and from 274 cases, patients without seizures were 156 (56.9%) who had abnormal brain MRI but noting the *P* = 0.44 significant difference has not been seen. Considering that 56% of affected children aged less than 2 years admitted before seizure stage, it may be possible that they suffer from seizures in the later stages of their maturity. Therefore, to achieve more eligible results, the later followup of these children should be evaluated.

Other important points of this study are about close relative parents of the affected children (56.9%). These findings in children with abnormal brain MRI are higher than those reported in normal brain MRI meaning that from 330 cases with relative parents 201 cases (60.9%) were assigned abnormal brain MRI where this finding between 250 children with nonrelative parents was only 39 cases (55.6%). Although this is statistically not considered as the significant difference, regardless of the brain MRI findings, and with regard to social context of the region, frequency of relative marriage, hereditary chromosomal disorders, and metabolic diseases as important causes of GDD, this result can be expected and emphasizes the necessity of identifying these diseases.

## Figures and Tables

**Table 1:** MRI findings in study groups based on age and gender variables.

Variables	MRI findings					
			No. (%)			
	Normal	Abnormal				
		1	2	3	4	5
Age (months)						
2–6	20 (35.7)	6 (10.7)	5 (8.9)	0 (0)	18 (32.1)	7 (12.5)
7–24	92 (33.7)	24 (8.8)	18 (6.6)	2 (0.7)	114 (41.8)	23 (8.4)
25–60	66 (46.5)	5 (3.5)	8 (5.6)	0 (0)	54 (38)	9 (6.3)
61–164	62 (56.9)	3 (2.8)	8 (7.3)	1 (0.9)	32 (29.4)	3 (2.8)

Total	240 (41.4)	38 (6.6)	39 (6.7)	3 (0.5)	218 (37.6)	42 (7.2)

Gender						
Male	129 (38.7)	22 (6.6)	23 (6.9)	2 (0.4)	137 (41.1)	20 (6)
Female	111 (44.9)	16 (6.5)	16 (6.5)	1 (0.4)	81 (32.8)	22 (8.9)

Total	240 (41.4)	38 (6.6)	39 (6.7)	3 (0.5)	218 (37.6)	42 (7.2)

1: Non-specific findings, 2: Congenital and developmental abnormalities; 3: Detectable syndromes, 4: Traumatic and neurovascular diseases, 5: Metabolic and degenerative diseases.
